# Leveraging the electronic health record to eliminate hepatitis C: Screening in a large integrated healthcare system

**DOI:** 10.1371/journal.pone.0216459

**Published:** 2019-05-23

**Authors:** Alexander G. Geboy, Whitney L. Nichols, Stephen J. Fernandez, Sameer Desale, Peter Basch, Dawn A. Fishbein

**Affiliations:** 1 MedStar Health Research Institute, Hyattsville, MD, United States of America; 2 MedStar Institute for Innovation, Washington, DC, United States of America; 3 MedStar Quality and Safety Institute, Washington, DC, United States of America; 4 MedStar Washington Hospital Center, Washington, DC, United States of America; University of New Mexico Health Sciences Center, UNITED STATES

## Abstract

Highly efficacious and tolerable treatments that cure hepatitis C viral (HCV) infection exist today, increasing the feasibility of disease elimination. However, large healthcare systems may not be fully prepared for supporting recommended actions due to knowledge gaps, inadequate infrastructure and uninformed policy direction. Additionally, the HCV cascade of care is complex, with many embedded barriers, and a significant number of patients do not progress through the cascade and are thus not cured. The aim of this retrospective cohort study was to evaluate a large healthcare system’s HCV screening rates, linkage to care efficiency, and provider testing preferences. Patients born during 1945–1965, not previously HCV positive or tested from within the Electronic Health Record (EHR), were identified given that three-quarters of HCV-infected persons in the United States are from this Birth Cohort (BC). In building this HCV testing EHR prompt, non-Birth Cohort patients were excluded as HCV-specific risk factors identifying this population were not usually captured in searchable, structured data fields. Once completed, the BC prompt was released to primary care locations. From July 2015 through December 2016, 11.5% of eligible patients (n = 9,304/80,556) were HCV antibody tested (anti-HCV), 3.8% (353/9,304) anti-HCV positive, 98.1% (n = 311/317) HCV RNA tested, 59.8% (n = 186/311) HCV RNA positive, 86.6% (161/186) referred and 76.4% (n = 123/161) seen by a specialist, and 34.1% (n = 42/123) cured of their HCV. Results from the middle stages of the cascade in this large healthcare system are encouraging; however, entry into the cascade–HCV testing–was performed for only 11% of the birth cohort, and the endpoint–HCV cure–accounted for only 22% of all infected. Action is needed to align current practice with recommendations for HCV testing and treatment given that these are significant barriers toward elimination.

## Introduction

The “silent” hepatitis C virus (HCV) epidemic is no longer silent: it is the most common blood-borne infection in the United States affecting between 2.7 and 5.2 million people [1–3] and is estimated to affect 71 million people worldwide [4]. It accounts for more deaths per year in the US than any other infectious disease including HIV [5], and only approximately half of those chronically HCV-infected have been diagnosed [6,7]. The prevalence of HCV among persons born in the Birth Cohort (BC) within 1945–1965 is five times higher than adults born in other years [8]. However, the incidence of acute HCV infections is rising in the non-BC among adolescents and young adults (aged ≥ 30 years) largely due to injection drug use [9,10].

To combat increasing morbidity and mortality associated with chronic HCV, in 1998 the Centers for Disease Control and Prevention (CDC) issued recommendations for risk-based HCV antibody (anti-HCV) testing targeting risk factors such as injection drug use (IDU) [11]. However, studies examining primary care provider testing practices revealed limited adherence, with screening rates in eligible patients ranging from 4.3% - 39.7% [12–17]. Limiting factors contributing to low uptake included the non-reporting by patients of current or historical risk, perceived irrelevance of risk-factor ascertainment to primary care visit reason, and a skepticism of the overall benefits outlined in the guidelines [7,8,12,18].

Recognizing the need for more inclusive guidance, in 2012 the CDC issued modified recommendations adding BC testing due to the high anti-HCV positive prevalence in this group [8]. In 2013, the United States Preventive Services Task Force (USPSTF) also concluded that there was ample evidence to issue a grade B recommendation for one-time testing for the BC and annual testing for adults at high risk for HCV infection [8,19]. Yet, despite the utility of these expanded guidelines, screening within larger primary care settings post 2012 has remained suboptimal with reported rates of 17.1% and 21.3% of eligible patients [20,21].

Concurrent with these testing recommendations, new HCV therapeutics were approved by the Food and Drug Administration (FDA) that are highly tolerable, all oral, and lead to cure rates of over 95%. The development of integrated and effective identification, screening, and linkage to care protocols within large health care systems is vital to uncovering the full burden of disease. Widespread HCV identification along with highly efficacious therapeutics renders HCV elimination feasible worldwide [22]. However, there remain considerable barriers to overcome in order to realize this goal, including incomplete disease surveillance, high cost of treatment, stigma, and lack of public health prioritization [23]. To this end, the HepC Testing and Linkage to Care program at MedStar Health Research Institute sought to leverage the Electronic Health Record (EHR) to identify and monitor HCV screening rates and institutional linkage to care proficiency within a large integrated healthcare system through the creation of a BC HCV clinical decision support (CDS) prompt. The aim of this study was to evaluate a large healthcare system’s HCV screening rates, linkage to care efficiency, and provider testing preferences. This study was approved by the MedStar Health Research Institute (MHRI) Institutional Review Board (IRB 2015–071).

## Materials and methods

### Study setting

MedStar Health (MSH) is the largest distributed care delivery network in the Maryland and Washington D.C. region, operating ten hospitals and supporting 184 outpatient care facilities throughout DC, Northern Virginia, Southern Maryland, and north of Baltimore, Maryland, serving a diverse patient population in urban, suburban and rural locations.

### Study population

A MSH database search identified approximately 750,000 persons in the system born between 1945 and 1965, 270,000 of whom had been seen between 2013–2015 in the outpatient system, and 11,500 who were known to have a diagnosis of HCV in the same time period. Applying the 3.25% MMWR BC estimate equates to over 24,000 persons infected with HCV within MSH.

Patients eligible to receive the CDS prompt included those born within 1945 and 1965, seen at a MSH primary care outpatient facility starting July 1, 2015 and who had at least one primary care visit during this time. During the period of July 1, 2015 through December 31, 2016, there were more than 230,000 primary care visits to a MSH facility. Demographics of the entire MSH population are approximately 60% women, 50% white and 35% black or African American, 3% Hispanic or Latino; 55% are privately insured while 36% have either Medicare or Medicaid.

Non-BC at-risk patients were not included in the CDS prompt although the prompt message read that all patients at risk should be tested. Patients were also excluded if they had previously been screened for, or diagnosed with, HCV infection as reported in the EHR.

### EHR protocol implementation and data abstraction

Data is reported from July 1, 2015 through December 31, 2016. During this study period, MSH primarily utilized the General Electric Centricity EHR for outpatient care documentation for both primary and specialty care clinic visits. In November of 2014, an automated CDS EHR-based HCV testing prompt for the BC was approved by the MedStar Ambulatory Quality Best Practices Committee. Its final design exclusively targeted the BC. Building such a prompt targeting non-BC and high-risk patients was determined to be beyond the capacity of the EHR platform as HCV-specific risk factors used to identify eligible patients for HCV testing (i.e., current or historical IDU, persons who received blood transfusions prior to 1992 [24] were not often captured, or if so they were captured as unstructured data. Non-BC testing outside the prompt was monitored, however, as a marker for potential at–risk patients, though these data are not reported.

**Fig 1:**
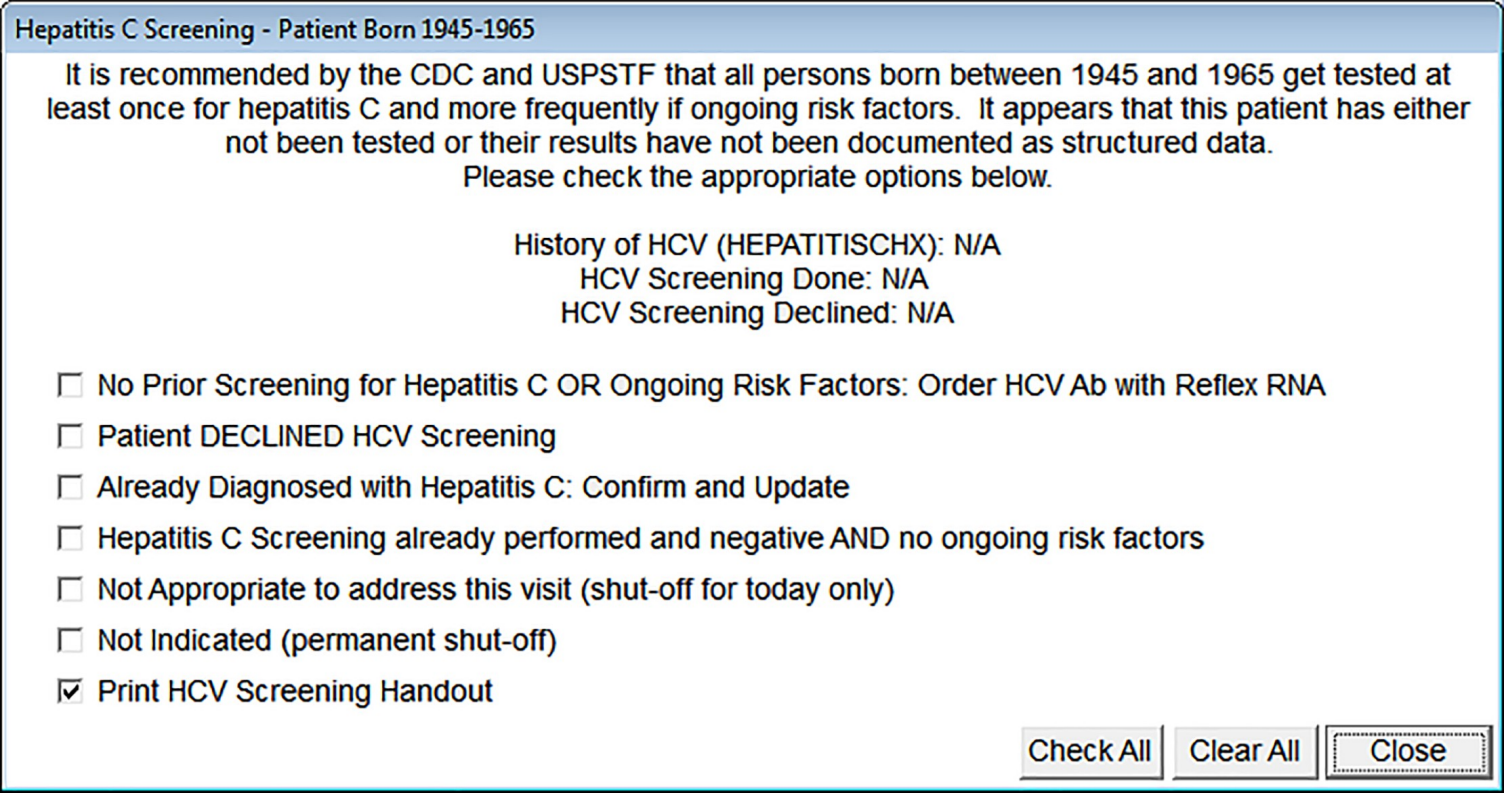
MedStar Health primary care centricity prompt, July 1, 2015 –December 31, 2016. Reprinted from MedStar Health Centricity EMR under a CC BY license, with permission from GE Healthcare, original copyright 2014.

**Fig 2:**
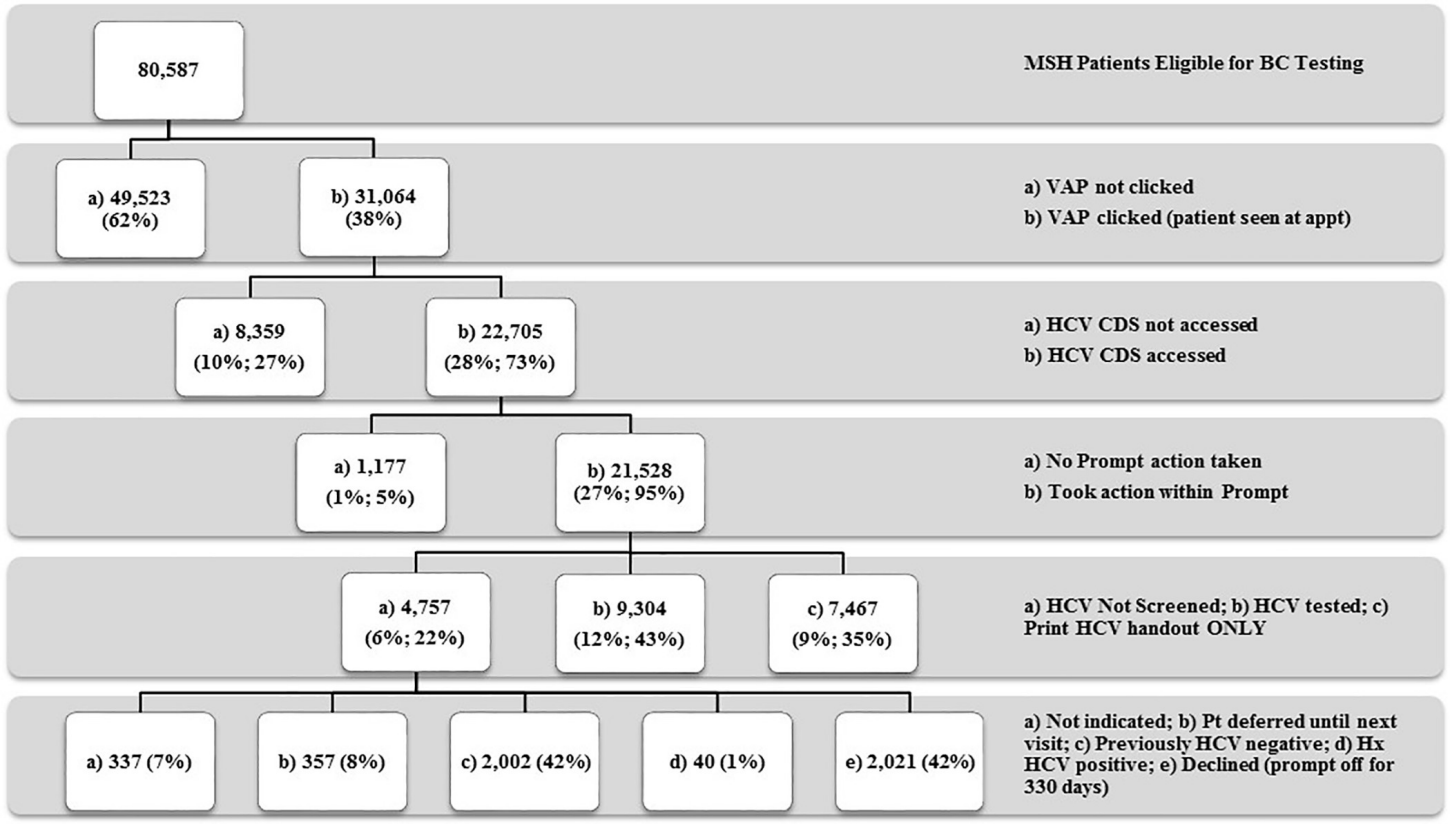
Clinical decision support: EHR clicks per actionable option.

The final CDS prompt was programmed to automatically populate given the above inclusion criteria. Once a patient was identified as eligible, the CDS was activated within the patient’s EHR chart. The CDS prompt was then triggered when a provider clicked the “View All Protocols” (VAP) yellow button containing health maintenance protocols and then checked the “Hepatitis C Screening” protocol option. Once checked, it appeared on-screen with seven discrete actionable options (Fig 1). The option to print a CDC HCV screening handout was automatically selected, thus encouraging testing and providing an educational resource. To test for HCV, a provider selected “*No Prior Screening for Hepatitis C OR Ongoing Risk Factors*: *Order HCV Ab with Reflex RNA*”, which then automatically populated a HCV antibody with reflex to verification NAA laboratory order and inserted the observation (OBS) term “HEPCAB” into a searchable, structured data field. When clicked, all actionable options produced a discrete, structured, and searchable OBS value within the EHR (Fig 2). The CDS prompt did not contain a hard stop, thus a provider was not required to open it or order a HCV screening lab. Furthermore, a provider was not prevented from bypassing it altogether, nor was it the only method to order an HCV test, which could be ordered at any point, without referring to the VAP yellow button.

Patient data were extracted from the EHR to monitor testing, prevalence and linkage to care. Patient demographics included date of birth, gender, race and ethnicity, insurance provider and home zip code. Other patient data included anti-HCV and HCV ribonucleic acid (RNA) results. Provider information included name, specialty, and clinic location. All test results were collected based on the location of the provider who signed for the laboratory result document to ensure test results were correctly allocated to the proper site.

### Statistical analysis

For this retrospective cohort study, descriptive statistics are presented. Chi-square was used to test the association between two categorical variables. Analysis was conducted using SAS version 9.4 (SAS Institute Inc., Cary, NC).

## Results

### Birth cohort CDS prompt metrics

Between July 2015 and December 2016, 80,556 patients across MSH initially met inclusion criteria as they were seen at an appointment and were considered eligible for BC HCV testing. Providers clicked the general VAP button for 31,064 (38.5%) individual eligible patients seen at a primary care appointment, accessed the HCV-specific CDS prompt for 22,705 patients (28.2% of those total eligible; 73.1% of those who clicked any VAP), and took an action within it for 21,528 patients (26.7% of those eligible; 94.8% of the CDS prompts accessed). Of these 21,528 patients, 9,304 (11.5% of those eligible; 43.2% of CDS prompt actions) were HCV tested through the CDS prompt and had a resulting laboratory value (Fig 2).

### Patient demographics

Of the 9,304 patients screened for HCV across MSH, the mean age was 59.8 ± 5.7 years, 35.7% (n = 3,323) had public health insurance (Medicare or Medicaid), 57.3% (n = 5,332) of patients were female, 42.4% (n = 3,948) were identified within the medical record as non-Hispanic, white, and 42.3% (n = 3,936) were identified as non-Hispanic, black or African American (b/AA) as shown in **Table 1**. It should be noted that full patient demographics for the entire 80,556 patient cohort were not obtained.

**Table 1:** Characteristics of patients tested across MedStar Health, July 1, 2015—December 31, 2016.

Characteristics	MedStar Health Prompt Denominator	Number of anti-HCV tests performed (percent)	Number of anti-HCV positive results (percent)	P-Value^a^	Number of HCV RNA-positive results (percent)	P-Value
**Total**	80,556 (100)	9,304 (11.5)	353 (3.8)		186/311 (59.8)	
**Age in years, mean ± standard deviation**	60.3 ± 5.8	59.8 ± 5.7	59.8 ± 5.7		59.8 ± 5.2	
**Sex**						
Female	48,086 (59.7)	5,332 (57.3)	135 (38.2)		63 (33.9)	
Male	32,463 (40.3)	3,972 (42.7)	218 (61.8)	*<0*.*0001*	123 (66.1)	*NS*
Undefined	7 (0)					
**Race/Ethnicity**						
Non-Hispanic white		3,948 (42.4)	109 (30.9)		43 (23.1)	
Non-Hispanic black/African American		3,936 (42.3)	195 (55.2)	*<0*.*0001*	115 (61.8)	*NS*
Latino/Hispanic		206 (2.2)	7 (2.0)		3 (1.6)	
Asian		170 (1.8)	-		-	
American Indian/Alaska Native		23 (0.3)	1 (0.3)		1 (0.5)	
Unspecified/Other		1,018 (11.0)	41 (11.6)		24 (13.0)	
**Health insurance**						
Private		5,685 (61.1)	139 (39.4)		61 (32.7)	
Public		3,323 (35.7)	203 (57.5)	*<0*.*0001*	121 (65.1)	*NS*
Medicare		1,886 (56.8)	82 (40.4)		46 (38.0)	
Medicaid		1,437 (43.2)	121 (59.6)	*<0*.*0001*	75 (62.0)	*NS*
Self Pay/Other		296 (3.2)	11 (3.1)		4 (2.2)	

^a^P-values calculated using Pearson’s χ^2^ test

### HCV infection and linkage to care

Regarding HCV positivity, 3.8% (n = 353/9,304) were anti-HCV positive, 89.8% (n = 317/353) received an HCV RNA lab order, 98.1% (n = 311/317) were HCV RNA resulted, and 59.8% (n = 186/311) were HCV RNA positive and thus HCV infected. Though more women than men were tested overall, men were twice as likely to test anti-HCV positive (OR 2.2 [1.8–2.8]). B/AA patients were more likely to test anti-HCV positive than all other groups combined (OR 1.7 [1.4–2.1]), as were b/AA men (OR 3.2 [2.5–4.0]). Patients with public insurance (i.e., Medicaid and Medicare) were more likely to test anti-HCV positive than those with all other insurance plans (OR 2.5 [2.0–3.1]) as seen in **Table 1**.

**Fig 3:**
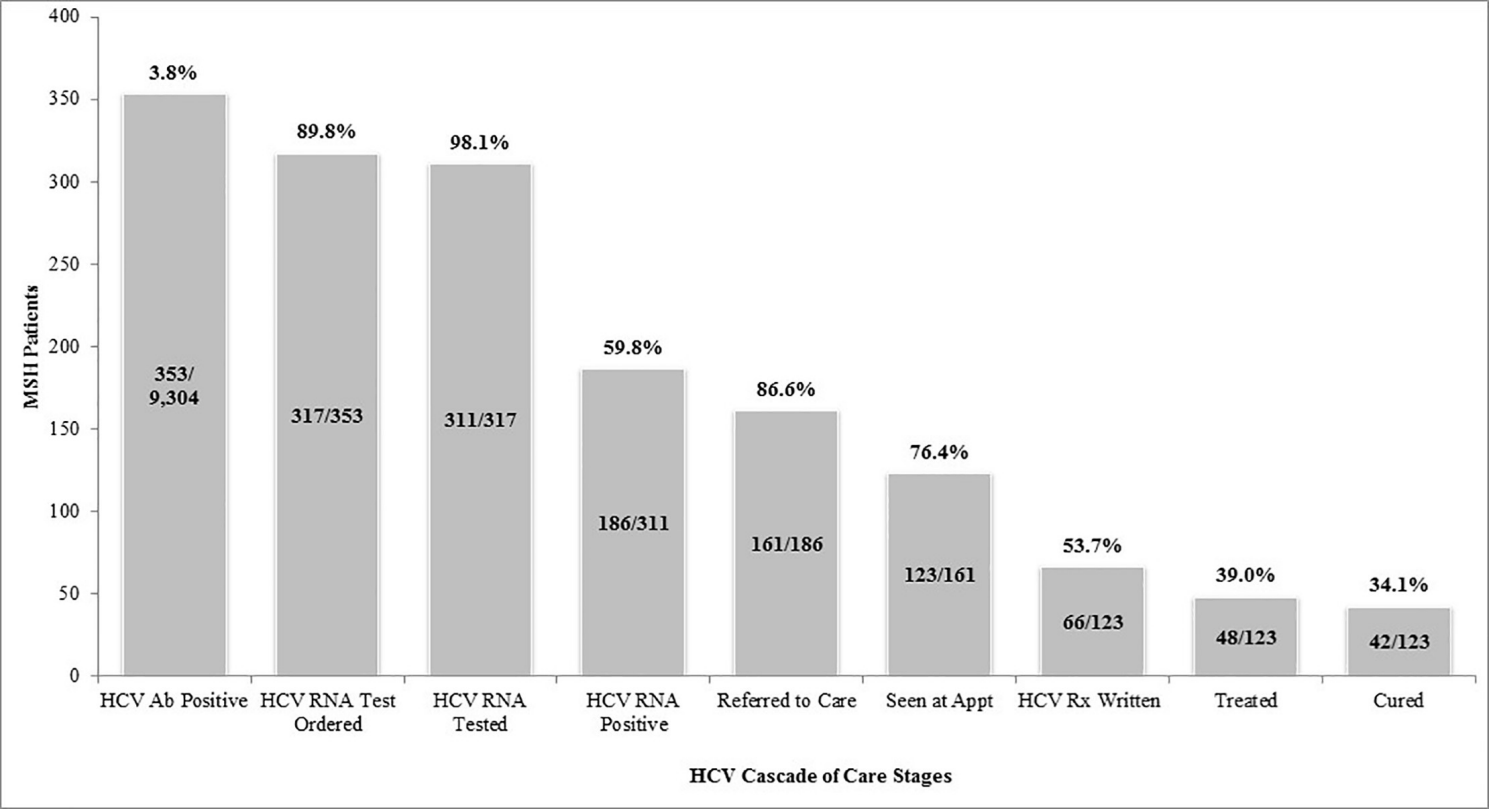
MedStar Health HCV cascade of care.

Regarding linkage to care and treatment, 86.6% (n = 161/186) of patients HCV RNA positive [infected] received a referral to a specialty provider (Gastroenterology, Hepatology, or Infectious Diseases), 76.4% (n = 123/161) of those referred were seen at the appointment, and 87.5% (42/48) who were treated were cured at the time of database close (Fig 3). All patients were treated with new DAA regimens.

### Testing distribution

Testing was conducted by approximately 682 providers within 141 primary care provider sites at MSH (standalone clinics, independent practices, or departments within a hospital/clinic setting) within Washington, D.C. (DC), Maryland (MD), and Virginia, with 83.7% (n = 7,786) of all tests being conducted by providers with an Internal Medicine specialty, 10.6% (n = 982) with Family Medicine specialty, and 5.8% (n = 536) tests distributed between 18 other departments, though providers ordering these tests were also located within primary care locations.

**Fig 4:**
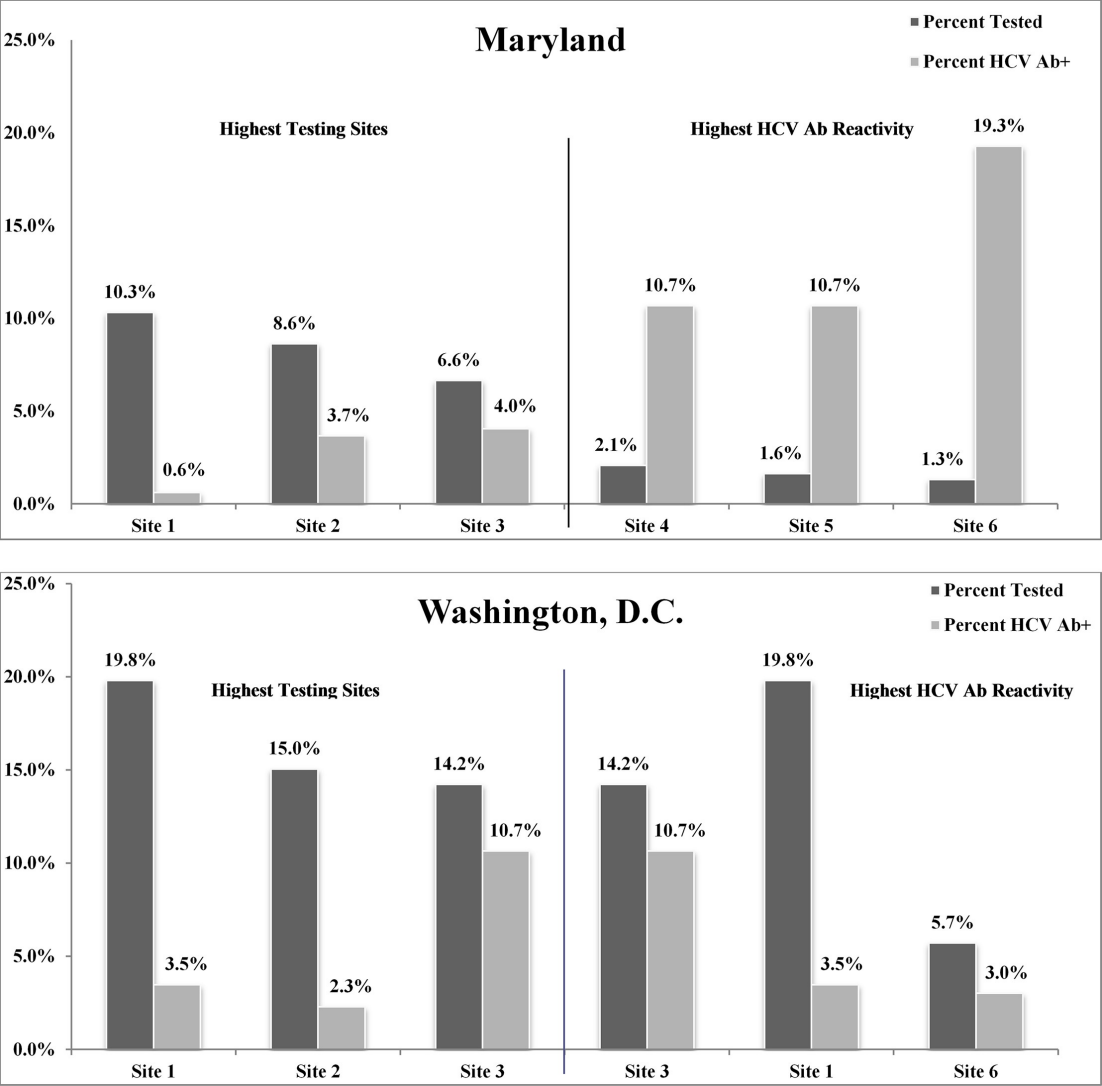
Top 3 highest testing sites compared to Top 3 Highest HCV Ab reactivity sites.

Maryland based provider sites (n = 83) accounted for 67.9% (n = 6,320/9,304) of all HCV Ab tests, with a MD-wide anti-HCV positive prevalence rate of 3.5% (n = 224/6,320). There was more diffuse reactivity throughout MD as compared to DC, with 50.6% of sites (n = 42/83) identifying at least one anti-HCV positive patient. Given this, 62.5% (n = 140/224) of anti-HCV positive tests were concentrated within 10 sites, each with at least 11 positive tests, located either within the city of Baltimore (8/10), or a neighboring county. Additionally, the highest testing site identified the lowest reactivity, while the lowest testing site identified the highest reactivity (Fig 4). In MD, those anti-HCV positive were more likely to be male than female (OR 2.1 [1.6–2.8]), b/AA men than white men (OR 1.5 [1.0–2.1]), and b/AA men than b/AA women (OR 2.8 [1.8–4.2]). There was no difference between total b/AA and white groups (*p* = 0.51).

Washington, DC based provider sites (n = 58) accounted for 31.2% (n = 2,904/9,304) of all HCV Ab tests, with a DC-wide anti-HCV positive prevalence rate of 4.4% (n = 128/2,904). Of the 58 sites, 34.7% (n = 24/58), were responsible for all 128 anti-HCV positive tests (Fig 4). In DC, those anti-HCV positive were more likely to be male than female (OR 2.4 [1.7–3.4]), b/AA than white (OR 6.3 [3.7–10.7]), and b/AA men than b/AA women (OR 3.8 [2.5–6.0]).

Comparing the two regions, though 67.9% (n = 6,320) of HCV tests were conducted in MD, DC had a higher overall reactivity rate (4.8% vs. 3.5%, *p =* 0.047). Comparing like groups (i.e., b/AA and from DC vs. b/AA and from MD), statistically significantly higher reactivity was observed in b/AAs overall (OR 2.2 [1.6–2.9]), in b/AA men (OR 2.6 [1.8–3.7]), and in b/AA women (OR 1.8 [1.1–2.9]) from DC, while statistically significantly higher reactivity was observed in white men (OR 3.3 [1.6–6.9]) and overall white groups (OR 2.6 [1.6–4.8]) from MD. Last, it should be noted that there were 66 HCV tests conducted in VA, with an overall anti-HCV positive prevalence rate of 1.5% (n = 1/66).

## Discussion

The 11.5% MSH-wide BC screening rate of eligible patients (n = 9,304/80,556), was higher than one recently reported study describing national trends in HCV BC antibody testing of 3.3% [25], but lower than similar studies conducted after the 2012 CDC guidelines were released, of 18.6% and 21.3% respectively [20,21]. Nonetheless, this rate is suboptimal considering BC screening recommendations are now well-established (since 2012) and Medicare reimburses a single HCV Ab test for this population. This rate likely reveals universal gaps in testing methodology, provider knowledge and behavior, and suggests provider-initiated testing, regardless of prompts, fall short of expectations.

Overall, anti-HCV positive cases (3.8%) across the MSH network were congruent with the CDC BC prevalence rate of 3.25%, which is likely reflective of MSH being representative of the US population [8]. However, within individual sites, reactivity ranged from a high of 19% to less than 1% for sites that identified at least one reactive test. Sites with high reactivity need to be investigated further to determine whether the overall clinic populations contained fewer eligible patients, or whether providers were testing only those patients they anticipated to be infected. Similarly, sites with lower reactivity should also be investigated to determine whether providers were perhaps screening healthier patients and those more compliant with general medical care. It also needs to be considered whether these sites have appropriate resources for navigating their patients through the HCV cascade of care toward a cure. Other significant overall trends were also consistent with the BC literature from urban settings (i.e., patients were more likely to be anti-HCV positive if they were male and b/AA).

The MSH HCV RNA infection rate of 59.8%(n = 186/311) is notably lower than the often-reported rates of approximately 75%-85% [8], however there does appear to be variability within the literature. In 2012–2014, the CDC sponsored a nation-wide hepatitis C testing and linkage to care initiative [26]. Individual grantees testing similar populations to the one described in this study (i.e., previously undiagnosed BC patients) saw results ranging from 62.2% [27] and 64.7% to 72.3% [28,29] and 73.9% [30]. Interestingly, a comprehensive review of the 14 grantees supporting BC testing noted that chronic infection was identified in 75.6% (n = 822/1088) of patients who received same-day RNA testing as compared to 66.2% (n = 675/1020) who did not [31]. While this is evidence for the necessity of same-day HCV RNA reflex testing, it also points to the difficulty in obtaining a true rate of chronicity. Though the literature does generally support greater clearance rates in the BC, perhaps in those with active underlying risk factors and thus continued risk of reinfection there is less clearance. Lastly, the higher than expected RNA negative rate could be a consequence of either a false-positive anti-HCV results, or evidence of past resolved HCV infection. In both cases, persons testing anti-HCV positive and HCV RNA negative and without identified recent or active risk factors are considered negative for current infection and would not need further HCV testing.

The MSH HCV RNA testing rate of 88.1% (n = 311/353) was comparable to other recent studies [21,27,29,32–35], though fell short of 100% threshold [36], which was anticipated as the imbedded active decision support CDS prompt was for a HCV Ab with reflex to NAA test. There were two primary reasons for not reaching 100%: a) reflex processing errors with the need for a frozen specimen and/or an additional tube of blood; and b) incomplete use of the CDS prompt. To the latter reason, it is possible providers utilized the CDS prompt only as a guide or reminder to review a patient’s chart, but ordered a different lab test which did not include the reflex test. Nonetheless, as this study was observing provider practices across the entire system and without study intervention, the 76.4% linkage to care rate (n = 123/161) was lower than two recently reported linkage to care rates of 85.0% [2] and 92.2% [27], and higher than others ranging between 38.7% - 69.9% [21,29,32,33,35,37].

MSH did appreciably better at maintaining patients through the cascade of care (Fig 3) than other studies utilizing more targeted interventions [35,38], with 53.7% (n = 66/123) of patients receiving a prescription for HCV therapy, and 87.5% (n = 42/48) of those treated completed treatment achieving sustained virologic response (SVR) equating to cure. At the time of database close, six remaining patients had not yet returned for their end of treatment visit, though all 42 patients who completed treatment and had a 12-week post-treatment follow-up visit did achieve SVR. Regarding access to treatment, new oral DAAs were still being FDA approved during the study period. Starting in 2013, all new treatments required prior authorization. However, payers did not have a well-organized prior authorization approval process, and clinicians did not have adequate administrative support for the process. Once payer processes were established, there were fibrosis, sobriety and prescribing clinician specialty restrictions.

Additional work is underway to (a) identify factors that may be facilitators or barriers to successful HCV care, (b) intervene within the new EHR (MedConnect) by providing embedded reminders and resources to improve testing and linkage, and (c) educate primary care providers on HCV recommendations and best practices through quality improvement initiatives such as educational interventions (e.g., surveys evaluating clinician knowledge, barriers and attitudes, audit/feedback cycles, webinar sessions with HCV experts). Site comparisons will also need to be evaluated more closely considering multiple factors (i.e., white men in MD were more likely to test anti-HCV positive than white men in DC). To this point, there was variability between MD and DC sites regarding the association between highest testing and highest HCV Ab reactivity. In MD, sites that accounted for the highest overall testing rates had the lowest reactivity, while other sites with the highest reactivity accounted for the lowest testing. Within DC, however, two sites accounted for both the highest overall testing rate as well as the highest reactivity (Fig 4). Understanding these trends could provide valuable direction for the creation and implementation of targeted interventions to both increase testing and provide appropriate HCV services.

This study has several limitations. Providers did not need to utilize the CDS prompt, in part or in full, to test patients for HCV. As indicated in Fig 2, for 61.5% of patients seen by a provider, the VAP button was never clicked. Identifying the subsequent actions taken from this group was beyond the scope of this study, although ordering an HCV test could not be ruled out as a possible action. Once a provider entered the available VAP prompts, it appears they were more likely to not only access the HCV CDS prompt (73%), but to take an action within it (95%) (Fig 2). However, as there was not a one-to-one concordance between prompt testing orders and overall tests, it is possible the HCV Ab screening rate for this study was underestimated. Additionally, the affiliated test, HCV Ab with Reflex to Qualitative NAA, was not unique to the prompt and could be ordered outside the prompt.

Second, testing outcomes were dependent on provider action and proper documentation. Though the CDS prompt was programmed specifically for HCV testing, methods for extracting data from the EHR and tracking its utilization within the EHR is imperfect. Though all structured health information is stored as searchable observation terms, it is sometimes the case that searchable information pertaining to providers and provider locations, hospital locations, documents and associated signing provider, ordered tests and test codes, and patient information is all stored or coded differently (and sometimes incorrectly) between each site, therefore complicating and limiting the data extraction process. Furthermore, information that is contained as unstructured data, either as free text in provider notes or HCV lab values that do not populate to searchable fields such as flowsheets, are unsearchable and information is “lost” (at least with the current technology) to external quantification.

Third, it is unclear whether this CDS prompt led to increased testing, as there was no comparator group. Currently, these testing data are representative of MSH’s baseline, at least within the BC. Near the conclusion of 2016, MSH transitioned from the GE Centricity to Cerner MedConnect EHR; however, an HCV prompt has not yet been created and prior testing data has not yet migrated though it is currently underway.

One of the greatest challenges to eliminating HCV is the barrier to initial testing and identification of previously untested persons at risk for HCV. Though this EHR prompt approximated the magnitude and distribution of HCV within the BC across a large healthcare system, it highlighted several critical system-wide gaps in provider knowledge and behavior, including subsequent care needs, and EHR utility. It is unclear the degree to which primary care providers’ disease-specific knowledge, or the underlying framework and utility of the CDS prompt and EHR itself were limiting factors as these were not explicitly measured. A more knowledgeable provider base is certainly a benefit to any health system, and in this case would have likely been more aware of identification, screening, and linkage to care recommendations; however, it cannot alone be a solution to CDS non-adherence. Additionally, while this active decision support prompt at least followed certain structural recommendations for workflow integration (i.e., it was delivered at the point of care, patient specific, clinically relevant, as automated as possible, and not a hard-stop prompt), it was underutilized.

The success of any CDS prompt is measured by positive changes in provider behavior and/or clinical outcome [39]. Given a useful, useable, and effective prompt, coupled with appropriate educational interventions such as provider in-services, HCV-infected persons could be identified and likely treated faster and in earlier stages of fibrosis, thus reducing morbidity and mortality from complications related to cirrhosis and hepatocellular carcinoma. While this CDS prompt saw incomplete usage, there was enough activity to identify a MSH-wide anti-HCV prevalence rate of 3.8% within the BC, and individual site rates as high as 20%. In terms of outcome, this is evidence enough to suggest that HCV has, and will continue to have, a significant negative impact on the health of the MSH community of patients. These results therefore demonstrate the need for a more dynamic approach to constructing a CDS prompt focused on both optimal workflow integration and provider education. Additionally, buy-in from hospital and health-system committees appears requisite to fully impact this epidemic. The MSH health systems–and health systems in general–ought to consider complete testing automation with built in same-day reflex HCV RNA testing. This would not only comply with CDC/USPSTF/CMS recommendations, but would support recommended actions by the National Academies of Science, Engineering, and Mathematics [22] in support of the ultimate goal of HCV elimination by 2030.

## Supporting information

S1 Dataset HepTLC data file. (XLSX)
